# Antagonistic peptide technology for functional dissection of CLE peptides revisited

**DOI:** 10.1093/jxb/erv284

**Published:** 2015-06-30

**Authors:** Nathan Czyzewicz, Mari Wildhagen, Pietro Cattaneo, Yvonne Stahl, Karine Gustavo Pinto, Reidunn B. Aalen, Melinka A. Butenko, Rüdiger Simon, Christian S. Hardtke, Ive De Smet

**Affiliations:** ^1^Division of Plant and Crop Sciences, School of Biosciences, University of Nottingham, Loughborough LE12 5RD, UK; ^2^Department of Biosciences, Section for Genetics and Evolutionary Biology, University of Oslo, N-0316 Oslo, Norway; ^3^Department of Plant Molecular Biology, University of Lausanne, CH-1015, Lausanne, Switzerland; ^4^Institute for Developmental Genetics, Heinrich-Heine University, D-40225 Düsseldorf, Germany; ^5^Centre for Plant Integrative Biology, University of Nottingham, Loughborough LE12 5RD, UK; ^6^Department of Plant Systems Biology, VIB, B-9052 Ghent, Belgium; ^7^Department of Plant Biotechnology and Genetics, Ghent University, B-9052 Ghent, Belgium

**Keywords:** CLE, IDA, peptide structure, peptide variants, root, small signalling peptides.

## Abstract

In the *Arabidopsis thaliana* genome, over 1000 putative genes encoding small, presumably secreted, signalling peptides can be recognized. However, a major obstacle in identifying the function of genes encoding small signalling peptides is the limited number of available loss-of-function mutants. To overcome this, a promising new tool, antagonistic peptide technology, was recently developed. Here, this antagonistic peptide technology was tested on selected CLE peptides and the related IDA peptide and its usefulness in the context of studies of peptide function discussed. Based on the analyses, it was concluded that the antagonistic peptide approach is not the ultimate means to overcome redundancy or lack of loss-of-function lines. However, information collected using antagonistic peptide approaches (in the broad sense) can be very useful, but these approaches do not work in all cases and require a deep insight on the interaction between the ligand and its receptor to be successful. This, as well as peptide ligand structure considerations, should be taken into account before ordering a wide range of synthetic peptide variants and/or generating transgenic plants.

## Highlight

Information collected using antagonistic peptide approaches can be very useful, but these approaches do not work in all cases and require insight on ligand-receptor interactions and peptide ligand structure.

## Introduction

Small signalling peptides are able to elicit a vast array of biological and physiological responses, allowing the plant to develop and adapt to changes in the surrounding environment (Czyzewicz *et al.*, 2013; Murphy *et al.*, 2012). In the *Arabidopsis thaliana* genome, over 1000 putative genes encoding small, presumably secreted, signalling peptides can be recognized (Lease and Walker, 2006). These small signalling peptides are mainly perceived by receptors, such as receptor kinases, and in the *A. thaliana* genome, over 600 genes encoding putative receptor-like kinase (RLK) proteins have been detected (Shiu and Bleecker, 2001*a*, *b*). However, to date, only a small portion of these putative small signalling peptides have been functionally characterized and few have been linked to a receptor (Butenko *et al.*, 2009; Czyzewicz *et al.*, 2013; Lee and Torii, 2012; Murphy *et al.*, 2012).

Small signalling peptides consist of usually <20 amino acids in their mature form and rarely >120 amino acids as a full-length precursor. Although there are hardly any data for most small signalling peptides, they are likely often present at very low (nanomolar range) physiological concentrations. Forward and reverse genetic approaches have been employed to study the biological function of genes encoding small signalling peptides. For example, CLAVATA3 (CLV3), a peptide regulating maintenance of plant stem cells, was identified in a forward genetic screen (Clark *et al.*, 1995; Fletcher *et al.*, 1999). The *clv3* mutants have an enlarged shoot apical meristem (SAM) and floral meristems, which generate supernumerary floral organs, suggesting a general role in regulating above-ground meristematic growth (Clark *et al.*, 1996). CLV3 belongs to the family of CLV3/EMBRYO SURROUNDING REGION-related (CLE) peptides, which consists of 31 members in *A. thaliana*. These peptides share a conserved 12–14 amino acid C-terminal domain that is proteolytically released and has been shown to function in various contexts, including shoot and root meristem development, nodulation, embryo and endosperm development, regulation of root architecture in response to nutrients, and vascular development (Araya *et al.*, 2014; Cock and McCormick, 2001; Fiers *et al.*, 2005; Fiume and Fletcher, 2012; Hirakawa *et al.*, 2008; Hobe *et al.*, 2003; Jun *et al.*, 2010; Lim *et al.*, 2011; Mortier *et al.*, 2010; Okamoto *et al.*, 2013; Reid *et al*., 2011; Stahl *et al.*, 2009). Genetic interaction studies suggested CLV3 to act as a small signalling peptide since mutations in the RLK encoding gene, *CLV1*, had a similar phenotype to *clv3* mutants and the overexpression phenotype of *CLV3* was lost in the *clv1* mutant background (Brand *et al.*, 2000). Indeed, the identification of the mature active CLV3 peptide and biochemical evidence for its interaction with CLV1 was confirmed almost a decade later (Ogawa *et al.*, 2008; Ohyama *et al.*, 2009). This example illustrates some of the difficulties in identifying the mature active form of small signalling peptides in plants and thereafter finding their receptors and/or interacting signalling partners.

One major obstacle in identifying the function of genes encoding small signalling peptides is the limited number of available loss-of-function mutants, since most have no useful T-DNA insertions, partly because small genes are less likely to be targeted by a T-DNA insertion. To complicate matters further, the functional redundancy of some small signalling peptides and RLKs can mask phenotypes when only one family member is successfully disrupted. Although some small signalling peptides have been discovered through screening of T-DNA or transposon insertion mutants—such as INFLORESECENCE DEFICIENT IN ABSCISSION (IDA), TAPETUM DETERMINANT1 (TPD1), CLV3, and CLE40 (Butenko *et al.*, 2003; Fletcher *et al.*, 1999; Hobe *et al.*, 2003; Yang *et al.*, 2003), new approaches and technologies are required to facilitate the functional analyses of genes encoding small signalling peptides and their putative corresponding receptor partners (Butenko *et al.*, 2014; Stes *et al.*, 2015).

**Fig. 1.**
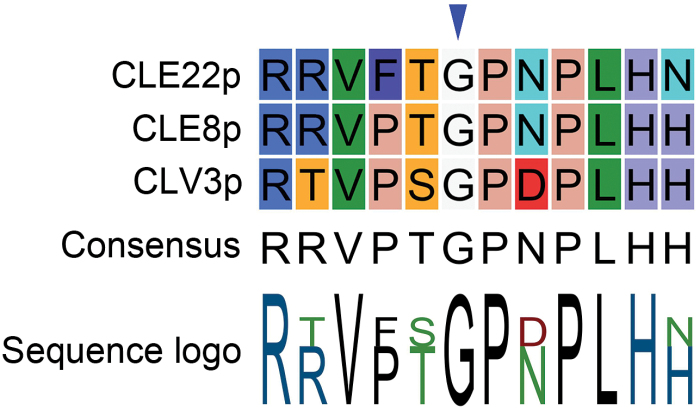
Alignment of CLE peptides used in Song *et al.* (2013). Conserved glycine (G) at position six is indicated with a blue arrowhead. (This figure is available in colour at *JXB* online.)

To interfere with and unravel endogenous peptide function, antagonistic peptides—such as mutant peptide variants, chemically modified peptides or peptide-like molecules that can affect peptide ligand–receptor (kinase) pathways are an important tool. In this context, structure-function/activity analyses can provide useful information on peptide residues critical for function. With respect to CLE peptides, such analyses were used to test, for example, suppression of nodulation capability in soybean (*Glycine max*) roots of the nodulation-controlling RHIZOBIA-INDUCED CLE1 (GmRIC1) (Reid *et al.*, 2013) or regulation of primary and lateral root growth of various CLE peptides (Czyzewicz *et al.*, 2015; Kondo *et al.*, 2008). Recently, this approach was used to develop a promising new tool, referred to as antagonistic peptide technology, for functional dissection of CLE peptides (Song *et al.*, 2013). Based on transgenic plants carrying CLV3 variants where each of the 12 residues in the core CLE motif were one by one replaced by alanine (Ala), it was shown that the glycine (Gly) to Ala substitution at position six gave a weak *clv3* phenotype. Subsequently, replacing this highly conserved Gly residue with other amino acids revealed that a Gly to threonine (Thr) produced a phenotype most similar to *clv3* mutants. This was further tested using synthetic CLV3 peptide with the Gly to Thr substitution (CLV3p^6Thr^), which was also able to produce—although less effective—the *clv3* mutant phenotype, and which could compete with wild-type synthetic CLV3 peptide (CLV3p). These exciting observations suggested that the CLV3p^6Thr^ variant could act as an antagonistic peptide. Specifically, a loss-of-function phenotype is suggested to be obtained through competitive inhibition, namely the peptide is proposed to be able to bind to the native receptor, but unable to activate it, since a functionally critical amino acid is mutated. Probably the CLV3p^6Thr^ variant has compromised peptide flexibility leading to stronger interaction with corresponding receptors and to disrupted downstream signal transduction. Taken together, such antagonistic peptides would provide a powerful tool for the functional dissection of CLEs in plants, and might also have the potential to be used for other plant peptides. Based on this assumption and the conserved nature of the Gly at position six (Fig. 1), this technology was applied to CLE8 (giving rise to embryo-lethal phenotype) and CLE22 (giving rise to short root phenotype) (Song *et al.*, 2013).

Here, this antagonistic peptide technology was tested, specifically Gly^6^-to-Ala or Gly^6^-to-Thr, as used by Song *et al.* (2013), on selected CLE peptides and the related IDA peptide, and its usefulness discussed in the context of studies of peptide function.

## Materials and methods

### Plant growth conditions

For the work on CLE40 and CLV3, seeds were surface sterilized with chlorine gas and imbibed in 0.1% (w/v) agarose for 2 d at 4 °C before being plated onto 0.5× Murashige and Skoog (MS) medium with Gamborgs No. 5 vitamins (Duchefa), 0.5g/l 2-(*N*-morpholino)ethanesulfonic acid (MES), 1% (w/v) sucrose, and 1.2% (w/v) plant agar. Plates were incubated vertically in a growth chamber with constant light at 21 °C for 5 d. For peptide-containing plates, synthetic dodecapeptides were added to a final concentration of 1 µM. For the work on CLE1/4, CLE7, CLE26, and CLE27, seeds were surface sterilized by immersion in 70% ethanol for 30 s, and incubated in 20% bleach at room temperature for 20min. Sterile seeds were vernalized in water at 4 °C for 2 d, before being plated onto 0.5× MS medium supplemented with 0.1g/l Myo-inositol (Sigma Aldrich), 0.5g/l MES (Sigma Aldrich), and 1% (w/v) bacteriological agar. Plants were incubated vertically under constant light at 21 °C until 12 d after germination. Synthetic CLE was added to a final concentration of 10 µM or 10nM. The work on CLE45 was essentially performed as previously described (Rodriguez-Villalon *et al.*, 2014).

### Starch staining

Starch granules and cell walls in root tips were stained with the mPSPI method and imaged with a confocal microscope as previously described (Truernit *et al.*, 2008).

### Oxidative burst experiments

For transient expression, *Agrobacterium tumefaciens* carrying HAESA-LIKE 2 (HSL2) in frame with eGFP in an estradiol-inducible expression vector described previously (Bleckmann *et al.*, 2010), was infiltrated into *Nicotiana benthamiana* leaves according to (Mueller *et al.*, 2012). The oxidative burst experiment was performed as previously described by (Butenko *et al.*, 2014), with the exception that 3 d after infiltration with *A. tumefaciens*, leaf pieces of *N. benthamiana* were induced with 20 μM estradiol before cut. Light emission was measured in a Wallac 1420 VICTOR^2^™ microplate luminometer (PerkinElmer).

### Peptide structure predictions

The recently published solution structure of CLE10p, solved using nuclear magnetic resonance (NMR) (MMDB ID: 125940; PMBID: 2MD), depicts the backbone of the PXGP core (position 4–7) as a smooth curve protruding from the rest of the peptide. To investigate the effect of mutations in this core of the peptides investigated, amino acid sequences with the structure AAA[core]AAA with the core PGGP, PGAP, PGTP, PRGP, PRTP, PSAP, or PSTP were submitted for analysis in PEP-FOLD (http://mobyle.rpbs.univ-paris-diderot.fr/cgi-bin/portal.py?form=PEP-FOLD#forms::PEP-FOLD) using standard settings.

## Results and discussion

### ‘Antagonistic’ CLE peptides

**Fig. 2.**
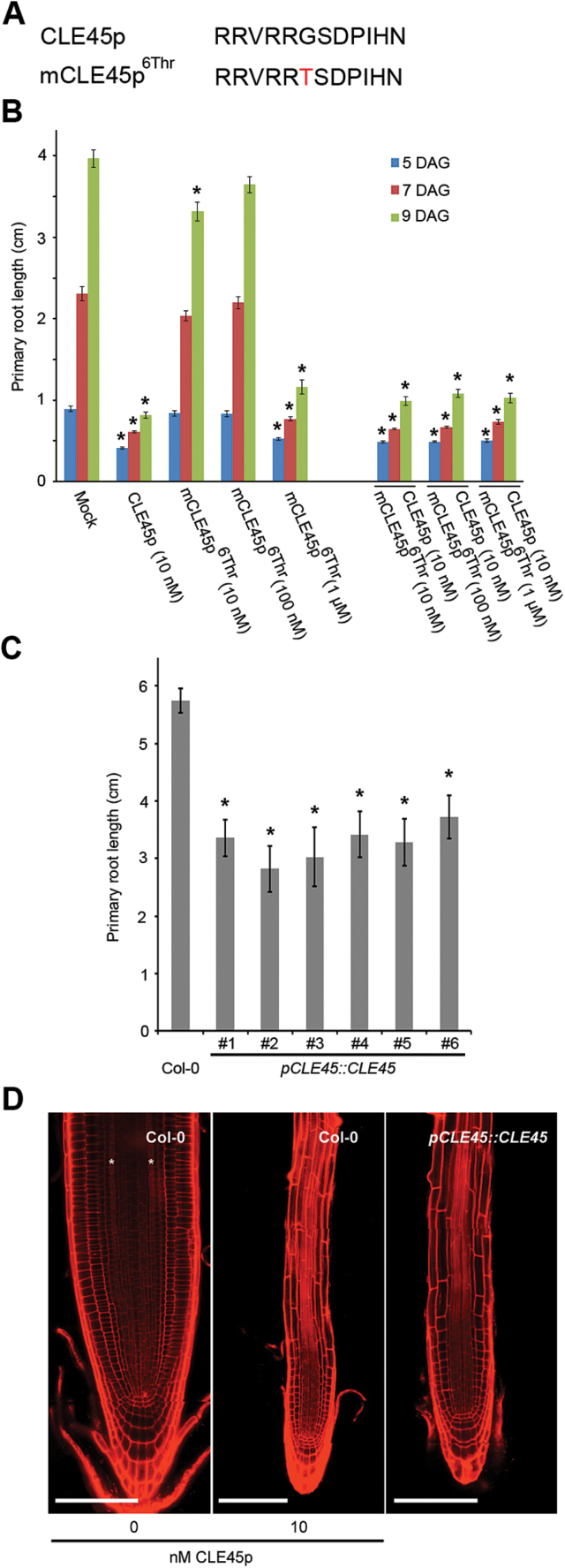
CLE45 peptide treatment and *pCLE45::CLE45* transgenic lines. (A) Sequence of synthetic CLE45 and mCLE45 peptides used. (B) Primary root length following treatment of wild-type seedlings with indicated concentrations of CLE45p or mCLE45p^6Thr^. The bar graph indicates the mean ± standard error. Statistical significance (Student’s *t*-test) compared with mock is indicated for each time point (DAG, days after germination): * *P* <0.01. (C) Primary root length of *pCLE45::CLE45* lines. The bar graph indicates the mean ± standard error. Statistical significance (Student’s *t*-test) compared with Col-0 is indicated: * *P* <0.01. (D) Confocal images of primary root meristems of 7-d-old seedlings (propidium iodide-stained; composite images). The asterisks highlight the two protophloem strands that can be distinguished in wild-type (Col-0) grown on mock (left), but that do not develop when grown on 10nM CLE45p (middle). Protophloem strands also do not develop in wild-type seedlings that express a *pCLE45::CLE45* transgene (right). Scale bar, 100 µm.

**Fig. 3.**
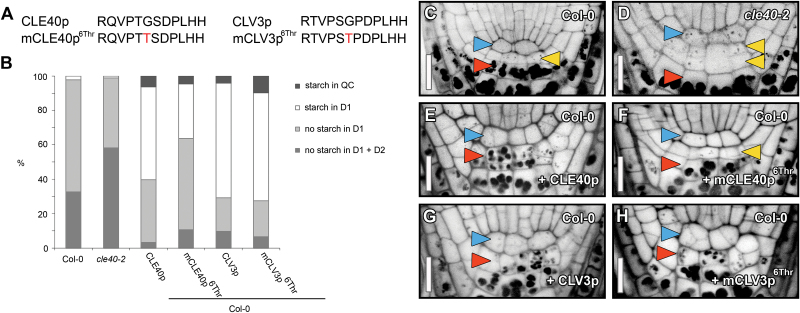
Distal root phenotypes after antagonistic peptide treatments. (A) Sequence of synthetic CLE and mCLE peptides used. (B–H) Distal root cell fates were analysed by mPSPI staining 5 d after germination in wild-type (Col-0) and *cle40-2* mutant roots (C, D). Representative examples of Col-0 roots grown on media with 1 µM CLE40p (E), mCLE40p^6Thr^ (F), CLV3p (G), and mCLV3p^6Thr^ (H) are shown. Frequency of roots carrying starch granules in the designated domains is shown in (B). Arrowheads: blue, QC position; yellow, CSC position (D1); red, CC position (D2). Double yellow arrowheads indicate CSC fate in D2, whereas the lack of a yellow arrowhead indicates CC fate in D1 position. QC, quiescent centre position; D1, distal layer position one; D2, distal layer position two; CC, columella cell position. Scale bars represent 15 µm.

**Fig. 4.**
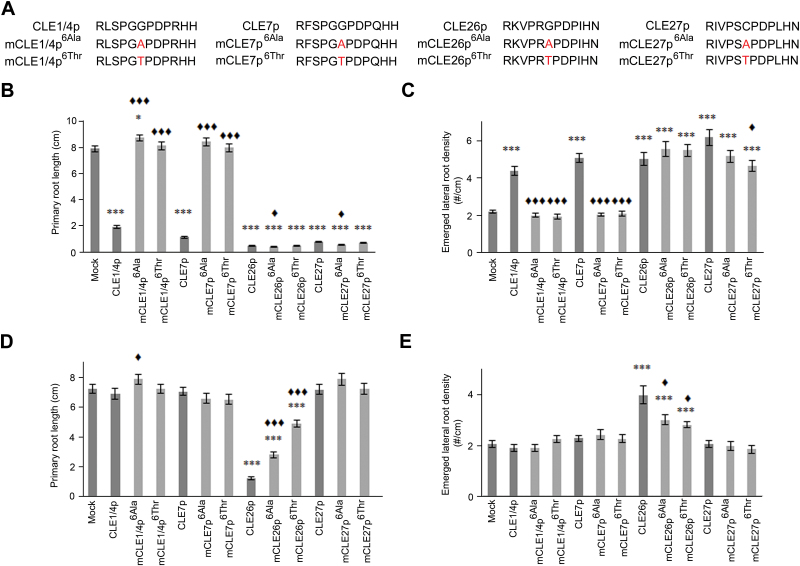
CLE1/4, CLE7, CLE26, and CLE27 peptide treatment. (A) Sequence of synthetic CLE and mCLE peptides used. (B–E) Treatment of wild-type seedlings with 10 µM (B, C) or 10nM of CLE or mCLE peptide (D, E). Quantification of primary root length (B, D) and emerged lateral root density (C, E) for CLEp and mCLEp-treated wild-type seedlings. The bar graph indicates the mean ± standard error. Statistical significance (Student’s *t*-test) compared with no peptide (*) and to CLEp treatment (^♦^) is indicated: ***/^♦♦♦^, *P* <0.001, */^♦^, *P* <0.05. (This figure is available in colour at *JXB* online.)

Among many processes (Cock and McCormick, 2001; Fiume and Fletcher, 2012; Hirakawa *et al.*, 2008; Okamoto *et al.*, 2013), various CLE peptides affect primary and lateral root growth and development (Czyzewicz *et al.*, 2015; Depuydt *et al.*, 2013; Fiers *et al.*, 2005; Hobe *et al.*, 2003; Jun *et al.*, 2010; Rodriguez-Villalon *et al.*, 2014; Rodriguez-Villalon *et al.*, 2015; Stahl *et al.*, 2009). To build on previous work investigating CLE peptides in the context of lateral root development, primary root growth, root apical stem cell maintenance, and vascular development, putative antagonistic versions of CLV3, CLE1/4, CLE7, CLE26, CLE27, CLE40, and CLE45 peptides were designed—based on the findings by Song *et al.* (2013)—to further unravel CLE peptide function (Figs 2A, 3A, 4A). To assess the function of these mutated chemically synthesized CLE peptides with Gly/cysteine (Cys) to Ala or Gly/Cys to Thr substitutions (referred to as mCLEp^Ala6^ or mCLEp^6Thr^, respectively), a number of biological assays were used.

The antagonistic peptide technology was first applied to CLE45 peptide (CLE45p), which, when applied exogenously, leads to shorter primary roots because it suppresses protophloem differentiation (Depuydt *et al.*, 2013; Rodriguez-Villalon *et al.*, 2014). To explore a potential loss-of-function phenotype, synthetic mCLE45p^6Thr^ peptide was applied and its effect on primary root development upon external application evaluated (Fig. 2A, B). This revealed that at the low nanomolar range mCLE45p^6Thr^ does not have an effect on primary root length as compared with the wild-type CLE45p (Fig. 2B), again confirming that position six is important for peptide activity. However, a higher concentration of 1 μM mCLE45p^6Thr^ had the same effect as the unmodified wild-type CLE45p (Fig. 2B). In addition, this peptide was not able to out-compete the effects of simultaneous CLE45p application (Fig. 2B). Thus, while the mCLE45p^6Thr^ peptide does not act as an antagonistic peptide, a CLE45p variant was obtained, which has identical effects as the wild-type version but required application of higher peptide concentrations. The notion that mCLE45p^6Thr^ is a weak CLE45p, rather than an antagonistic version, was confirmed *in planta* (Rodriguez-Villalon *et al.*, 2014). Plants that express a wild-type *pCLE45::CLE45* transgene are notoriously difficult to create, presumably because of the detrimental effects of increased CLE45 dosage. However, the few lines that were eventually obtained recapitulated the root phenotype observed upon external CLE45p application (Fig. 2C). Specifically, in *pCLE45::CLE45* lines, root growth was impaired, the periclinal division of the sieve element precursor cell was frequently abolished, and protophloem differentiation was often suppressed (Fig. 2D). This phenotype is similar to plants that express a corresponding *pCLE45::CLE45^6Thr^* transgene, which are much easier to obtain (Rodriguez-Villalon *et al.*, 2014). Thus, the data for both tissue culture assay and *in planta* are consistent with the interpretation that mCLE45p^6Thr^ is a weak rather than an antagonistic version of the CLE45 peptide.

Next, the antagonistic peptide approach for CLE40 was explored (Fig. 3A). It was previously shown that an increasing concentration of synthetic CLE40 peptide reduces stemness and causes differentiation of columella stem cells (CSCs), quiescent centre (QC) cells, and proximal initial (P1) cells in wild-type roots (Fig. 3B, D, H, Supplementary Table S1 available at *JXB* online) (Stahl *et al.*, 2013). Also synthetic CLV3 peptide acts similarly on the stemness in the root tip (Fig. 3E, G, Supplementary Table S1 available at *JXB* online). In contrast, in the shorter *cle40* mutant roots, differentiation of CSC daughters into CCs was significantly delayed (Fig. 3B, F, H, Supplementary Table S1 available at *JXB* online) (Hobe *et al.*, 2003; Stahl *et al.*, 2009). Wild-type roots carry mostly one (at D1 position) or, after a recent cell division, two layers of CSCs distal to the QC (at D1 and D2 positions), which lack stainable starch granules (Fig. 3B, H, Supplementary Table S1). In *cle40* root tips, additional CSCs in more distal positions (D2) were found (Fig. 3B, C, H, Supplementary Table S1). To assess if synthetic mCLE40p^6Thr^ and mCLV3p^6Thr^ variants could be used as antagonistic peptides to obtain a loss-of-function phenotype, their impact on the distal root stemness was evaluated (Fig. 3A). This revealed a response comparable with the wild-type CLE40p or CLV3p treatments (Fig. 3E, G, H, Supplementary Table S1 available at *JXB* online). Taken together, this suggests that the Gly to Thr substitution in CLE40 and CLV3 does not give rise to an antagonistic peptide.

Finally, while treatment of *A. thaliana* seedlings with 10 µM wild-type CLE1/4p, CLE7p, CLE26p, and CLE27p resulted in a short primary root and more lateral roots (Fig. 4B, C) (Czyzewicz *et al.*, 2015; Depuydt *et al.*, 2013; Kinoshita *et al.*, 2007; Rodriguez-Villalon *et al.*, 2014), this does not necessarily reflect their natural function. However, based on the *CLE1/4*, *CLE7*, *CLE26*, and *CLE27* expression patterns, a role in lateral root development might be expected (Czyzewicz *et al.*, 2015; Jun *et al.*, 2010). In this context, only CLE26p gave rise to a short primary root and increased lateral root density at a concentration of 10nM (Fig. 4B) (Czyzewicz *et al.*, 2015; Rodriguez-Villalon *et al.*, 2015), further supporting caution when interpreting exogenous peptide application results, especially at higher concentrations. To assess if the above-mentioned CLE peptides have a role in primary and lateral root development, the antagonistic peptide technology was attempted (Fig. 4A). However, analysis of mutated chemically synthesized CLE peptides (mCLEp) at 10 μM revealed that, although mCLE1/4p^6Ala/Thr^ and mCLE7p^6Ala/Thr^ did not induce a primary root shortening or a lateral root density increase—unlike the non-mutated forms of these peptides, mCLE1/4p^6Thr^ and mCLE7p^6Ala/Thr^ were also unable to produce an obvious dominant negative root phenotype, namely an expected longer primary root and/or decrease in lateral root density (Fig. 4B, C). However, for mCLE1/4p^6Ala^, a subtle increase in primary root length, but no effect on lateral root density was observed (Fig. 1C). It should be pointed out that since the receptor, and the associated loss-of-function phenotype, for these peptides is not known, the expected dominant negative root phenotype remains speculative. Nevertheless, this outcome suggested that for CLE1p, CLE4p, and CLE7p activity, the Gly at position six is essential, but that this mutant form did not appear to act as an antagonistic peptide. In contrast, mCLE26p^6Ala/Thr^ and mCLE27p^6Ala/Thr^ displayed a similar phenotype to the non-mutated forms, namely a significant reduction in primary root length (92–95%) and increased lateral root density (110–151%) (Fig. 4B, C), suggesting that the sixth amino acid in their respective sequences is not critical to their function, and also, did not appear to give rise to an antagonistic peptide when mutated. Intriguingly, at 10nM, mCLE26p^6Ala/Thr^ retained activity, but was less potent than CLE26p. This suggests that mCLE26p^6Ala/Thr^ is a weak rather than an antagonistic version of the CLE26 peptide, which is in agreement with the results on CLE45. In contrast, most mCLE1/4p, mCLE7p, and mCLE27p variants had no altered activity compared with the wild-type variant at 10nM, except mCLE1/4p^6Ala^ (Fig. 4D, E). In general, it appears that also for CLE1/4p, CLE7p, CLE26p, and CLE27p, the antagonistic peptide technology is not easily applicable.

In conclusion, other amino acid mutations are likely required to give rise to (strong) antagonistic CLE1p, CLE4p, CLE7p, CLE26p, CLE27p, CLE40p, and CLE45p peptides, or alternatively, this approach cannot be universally applied with respect to synthetic CLE peptides. A poor effect of synthetic antagonistic peptides could be due to delivery to relevant tissues and/or instability. However, synthetic peptide stability issues were not observed in these assays or with respect to synthetic control peptides, nor was a lack of phenotypes observed when synthetic (antagonistic) peptides were exogenously applied to the root. While the latter can be a non-specific effect in some cases, specific and local phenotypes were also observed.

### ‘Antagonistic’ IDA peptides

**Fig. 5.**
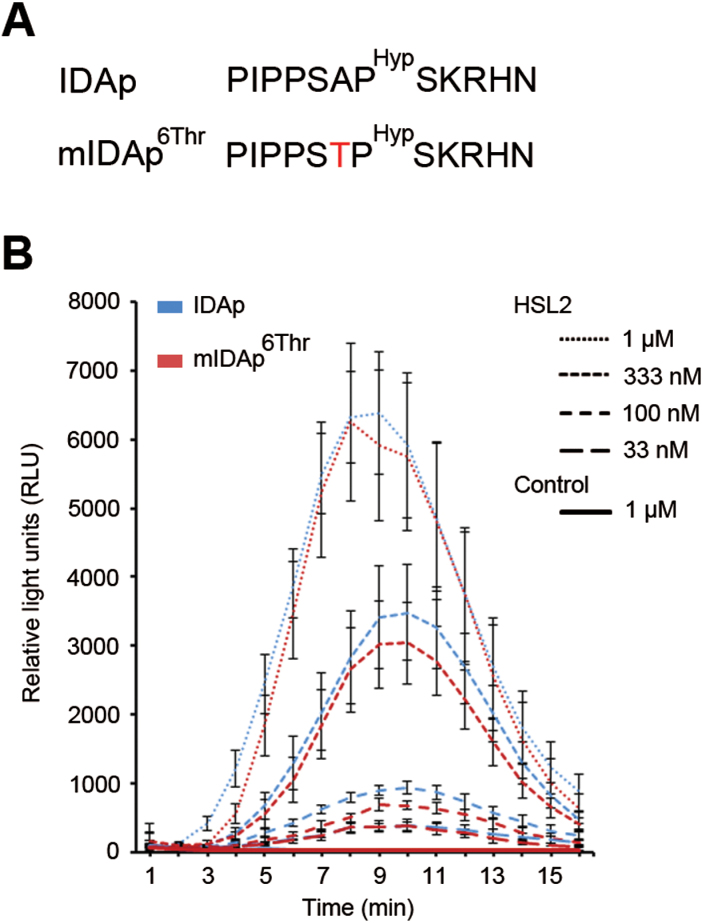
IDA peptide treatment. (A) Sequence of synthetic IDA peptides used. (B) *N. benthamiana* leaf pieces expressing *HSL2* were exposed to various concentrations of peptides as indicated. Oxidative burst by the luminol-based assay was monitored over time as relative light units (RLU). Leaf pieces infiltrated with *Agrobacterium* without HSL2 were exposed to 1 μM of both peptides and used as control. Error bars indicate standard error of *n*=3 or 4 replicates.

**Fig. 6.**
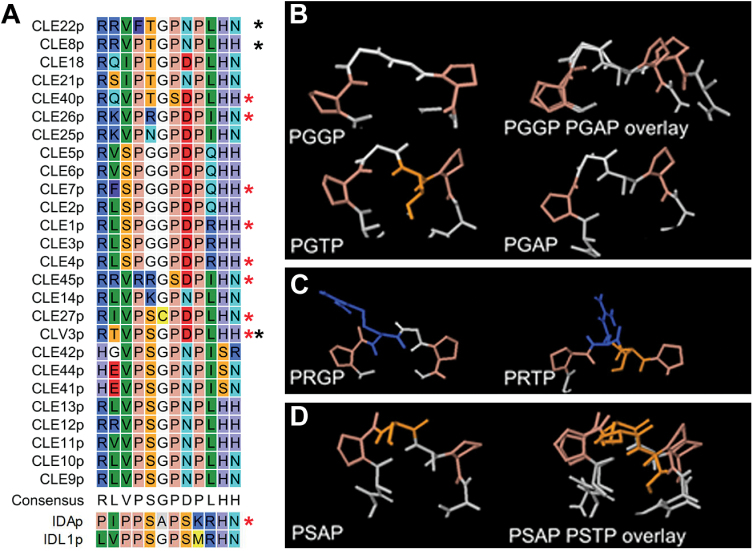
Peptide structure. (A) Manually adjusted alignment of the 12 amino acids of CLE peptides, with IDA and IDL1 for comparison. Red stars mark peptides used in the present study, black stars those tested by Song *et al.* (2013). (B–D) Examples of structures of the PXXP core predicted by PEP-FOLD. (B) PGGP. The larger Thr might interfere with receptor binding, and substitution of Gly6 with Ala6 may change the angles between the Pro residues. (C) PRGP. The side chain of Arg may change direction when the Gly6 is exchanged with a Thr. (D) PSAP. A change from Ala to Thr in position six may not result in major conformational changes when Ser is present in the core. (This figure is available in colour at *JXB* online.)

In addition, the extent the antagonistic peptide technology can be applied to other small signalling peptides was assessed. For this, the IDA and IDA-LIKE (IDL) family were chosen, given their sequence similarity to CLEs (Stenvik *et al.*, 2006). The IDA and IDL1 peptides of 12 amino acids share a common core at positions four to seven [PS(G/A)P] and the C-terminal end [H(N/H)] with CLV3 and some CLE peptides (Figs 5A, 6A). Like CLV3, hydroxylation of the Pro at position seven of the IDA dodecapeptide (IDAp, also referred to as PIPPo) increases the activity of the peptide (Butenko *et al.*, 2014). An oxidative burst response in *Nicotiana benthamiana* can be employed as readout for the RLK HAESA-LIKE2 (HSL2) activation by exogenously applied synthetic IDA peptides (Butenko *et al.*, 2014). Previous results indicated that IDAp binds to HSL2 with a Kd of 20nM (Butenko *et al.*, 2014). As the wild-type IDA peptide has an Ala at position six corresponding to the Gly at that position in CLV3, and the *ida* mutant phenotype can be fully rescued by IDL1, which has a Gly at this position (Stenvik *et al.*, 2008) (Fig. 6A); both of these small amino acids are evidently compatible with high signalling activity. It was, however, conceivable that substitution to the larger Thr (mIDAp^6Thr^) (Fig. 5A) could have an effect on receptor binding and/or activation. Therefore, the activity of mIDAp^6Thr^ in comparison with the activity of synthetic IDAp was assessed in an oxidative burst assay. For all peptide concentrations tested, mIDAp^6Thr^ gave the same response as IDAp in the presence of its receptor HSL2 (Fig. 5B), indicating that the mutated peptide was just as active as its wild-type counterpart. In conclusion, this mutation neither produced a ligand with weaker activity, nor a peptide with antagonistic effect.

## Conclusion

Information collected using antagonistic peptide approaches (in the broad sense) can be very useful, but these approaches do not work in all cases and require a deep insight on the interaction between the ligand and its receptor to be successful. While the antagonistic peptide approach might work in a number of cases, as described by Song *et al.* (2013) and Xu *et al.* (2015), its universal applicability remains to be determined. Initial data were presented for CLV3, CLE8, and CLE22, and recently for CLE19 but in the absence of the pertinent wild-type control transgenes and genetic knockout lines, it remains difficult to judge whether the phenotypes triggered by *mCLE8* ^*6Thr*^, *mCLE19* ^*6Thr*^, and *mCLE22* ^*6Thr*^ transgenes are antagonistic or not. Importantly, in view of the results presented here, and in agreement with the results of Song *et al.* (2013), it appears that the antagonistic peptide technology cannot be easily applied to synthetic CLE peptides and—at least—requires expressing mutant variants to deliver dominant peptides to their endogenous locations. However, as was shown with the *CLE45* ^*6Thr*^ transgene, the latter also does not always work. Nevertheless, it can provide novel insight that can lead to other tools to dissect peptide activity, as— for example—the weakened activity of mCLE45^6Thr^ could be used to functionally characterize CLE45. In addition, it also does not appear to be straightforward to translate this approach to other peptide families, as exemplified through analyses on IDA. In general, it was observed that whether the mutations have an effect or not, seems dependent on the context, with differential sensitivity to conformational changes (Fig. 6A and Supplementary Table S2 available at *JXB* online). CLE1/4p and CLE7p are highly similar peptides with the same PGGP core at position four to seven, and both lose activity when the Gly at position six is mutated to Ala or Thr. Structure prediction for the peptides may suggest that a mutation in this context, with the small Gly at position five, easily changes the peptide conformation (Fig. 6B). Alternatively, all size increases in the side chain of the amino acid at position six could interfere with binding of the putative receptor(s) of CLE1, CLE4, and CLE7. CLE26p and CLE45p both have an Arg in the core sequence (PRGP and RRGS, respectively) and react similarly to the introduced mutations, namely weaker activity when the Gly at position six is mutated. The long side chain of Arg might change direction in the mutant peptides, which might reduce its binding affinity for a receptor (Fig. 6C). In contrast, mutation of Ala at position six to Thr did not reduce the activity of the IDA peptide, which has a PSAP core, suggesting that the serine (Ser) residue might stabilize the peptide structure (Fig. 6D).

In conclusion, the antagonistic peptide approach can be a useful tool to study the function of some *CLE* genes (Song *et al.*, 2013; Xu *et al.*, 2015), but not the ultimate means to overcome redundancy or lack of loss-of-function lines (Rodriguez-Villalon *et al.*, 2014; this study). However, while the approach described by Song *et al.* (2013), when applied to synthetic CLE peptide variants, did not work—for the peptides selected in this study and with respect to the phenotypes investigated, it does not preclude there being any other substitution, modification, or combination thereof or a transgene that may induce the desired effects. This, as well as structure considerations, should be taken into account before ordering a wide range of synthetic peptide variants and/or generating transgenic plants.

## Supplementary data

Supplementary data are available at *JXB* online.

Supplementary Table S1. Quantification of distal root phenotypes after antagonistic peptide treatments.

Supplementary Table S2. Summary of mutations and phenotypes.

Supplementary Data
