# Supplementary material for: Antagonistic peptide technology for functional dissection of CLE peptides revisited
Source: J Exp Bot. 2015 Jun 30;66(17):5367–74. doi: 10.1093/jxb/erv284 (PMC4526918; doi:10.1093/jxb/erv284)
Supplement: Supplementary Data [file supp_66_17_5367__index.html]

Antagonistic peptide technology for functional dissection of CLE peptides revisited — Antagonistic peptide technology for functional dissection of CLE peptides revisited — Supplementary Data 

# Antagonistic peptide technology for functional dissection of CLE peptides revisited

## Supplementary Data

Data files

- Supplementary Data - Supplementary Data
