# Supplementary material for: Antagonistic peptide technology for functional dissection of CLE peptides revisited
Source: J Exp Bot. 2015 Jun 30;66(17):5367–74. doi: 10.1093/jxb/erv284 (PMC4526918; doi:10.1093/jxb/erv284)
Supplement: Supplementary Data [file supp_erv284_jexbot146936_file001.pdf]

SUPPLEMENTAL TABLES

	starch in D1	no starch in D1	no starch in D1+D2	starch in QC	starch in P1	n
**Col-0**	2.20	65.20	32.60	0.00	0.00	89
cle40-2	1.20	40.70	58.10	0.00	0.00	86
**Col-0 + CLE40p**	53.97	36.51	3.17	6.35	0.00	63
**Col-0 + CLE40p^{6Thr}**	31.82	53.03	10.61	4.55	0.00	66
**Col-0 + CLV3p**	66.67	19.44	9.72	4.17	0.00	72
**Col-0 + CLV3p^{6Thr}**	62.90	20.97	6.45	9.68	0.00	62

Table S1. Quantification of distal root phenotypes after antagonistic peptide treatments. Frequency in percent of *A. thaliana* roots carrying starch granules in the designated domains with or without peptide treatment. Seedlings were assayed by mPSPI staining 5 days after germination on medium ± 1 µM of peptide. P1 = proximal layer position one; QC = quiescent center position; D1 = distal layer position one; D2 = distal layer position two; n = number of main root tips analysed.

**Name**	**Sequence**	**6A**	**6T**
**CLV3p**	RTVPSGPDPLHH	nd	++
**IDAp**	PIPPSAPGRKHN	++	++
**CLE40p**	RQVPTGSDPLHH	nd	++
**CLE27p**	RIVPSCPDPLHN	++	++
**CLE26p**	RKVPRGPDPIHN	++	+
**CLE1/4p**	RLSPGGPDPRHH	-	-
**CLE7p**	RFSPGGPDPQHH	-	-
**CLE45p**	RRVRRGSDPIHN	nd	(+)

Table S2. Summary of mutations and phenotypes. Mutations at position six in CLE or IDA peptides has little effect in the context P[S/T]mP (where m is a mutation to A or T), but produce a peptide with reduced activity in the context [P/R]RmP. nd, not determined.
